# Macro- and microscopic changes in veins with short-term central venous catheters: an observational autopsy study

**DOI:** 10.1186/s12871-023-02380-x

**Published:** 2024-01-02

**Authors:** Mika M. Rockholt, Leila Naddi, Ahmed M. Badri, Elisabet Englund, Thomas Kander

**Affiliations:** 1 Department of Intensive and Perioperative Care, Skåne University Hospital, 221 85 Lund, Sweden; 2 Department of Anesthesiology, Perioperative Care and Pain Medicine, NYU Langone Health, NYC, NY USA; 3 Department of Clinical Sciences, Lund University, Box 117, 221 00 Lund, Sweden; 4 Department of Anaesthesiology and Critical Care, King Abdulaziz University Hospital, Jeddah, Saudi Arabia; 5 Department of Genetics, Pathology and Molecular Diagnostics, Region Skåne, Sweden

**Keywords:** Central venous catheterization, Complications, Central venous catheter thrombosis, Vascular Endothelium, Vascular injury

## Abstract

**Background:**

Centrally inserted central catheters (CICCs) are indispensable in modern healthcare, but unfortunately, come with complications. Catheter-related thrombosis is a well-known complication reported to occur in 5–30% of patients with CICC. There is a paucity of studies that report the incidence of catheter-related thrombosis after the introduction of real-time ultrasound insertion guidance as clinical practice. This study aimed to demonstrate any pathological macro- or microscopic changes in the vein wall associated with CICCs.

**Methods:**

The study was approved by the Swedish Ethical Review Authority and was conducted at a large university hospital. The study included 12 patients with a short-term CICC who were subject to autopsies. Vessels with inserted catheters were macroscopically and microscopically examined.

**Results:**

In total, seven female and five male patients with a median age of 70 (interquartile range 63–76) were included. With one exception, all patients received routine thromboprophylaxis throughout the period with CICC. Most inserted CICCs were 9.5 French (54%) and were inserted in the internal jugular vein (92%). The median time with CICC was seven days (interquartile range 1.8–20). At autopsy, thrombi were observed in all cases (100%), macroscopically and microscopically, attached to the distal portion of the CICC and/or the adjacent vessel wall. Inflammatory changes in the vessel walls were seen in all cases, and varying degrees of fibrosis were demonstrated in eight cases (67%).

**Conclusions:**

This autopsy study demonstrated that catheter-related thrombus formation with adjacent inflammatory and fibrotic vessel wall thickening was very common, despite a limited period of catheter use. The consequences of these findings are important, as thrombi may cause pulmonary embolism and possibly lead to catheter-related infections, and since inflammatory and fibrotic vessel wall thickening may evolve into chronic venous stenosis. Furthermore, the findings are a cause of concern, as CICCs are indispensable in modern healthcare and complications may be masked by the general disease that was the indication for CICC insertion.

## Background

In recent decades, numerous studies have assessed the incidence and risk factors for thrombosis associated with medical devices such as centrally inserted central catheters (CICCs) [1–5]. These indwelling catheters are indispensable in clinical settings, and modern healthcare cannot be provided without them.

However, as soon as a foreign biomaterial such as a CICC is introduced to the circulatory system, the host’s defence systems, including inflammation and coagulation, are activated, and cause the formation of a fibrin containing sleeve around the CICC [3, 6–8]. The sleeve normally evolves into a fibroblastic sleeve with subsequent collagen deposition but may also progress to a catheter-related thrombosis (CRT) adherent to the vein wall [1, 9]. The CRT may cause pulmonary embolism and possibly lead to catheter-related infections [4, 9, 10]. The pathological process is multifaceted and related to different risk factors, including endothelium damage [11], which also can lead to central vein stenosis [12]. Both the local endothelial injury at the insertion site and the more general endothelial dysfunction that comes with some of the indications for CICC, such as sepsis and cancer, may be important [13]. Together with blood congestion, which will occur when the thrombosis evolves, this completes the well-described Virchow’s triad [14].

The incidence of CRT in living patients varies in different publications from 5% for symptomatic events to 14–30% [5, 15–17]. Among these, the recently published study by Wu et al. should be highlighted [5]. In this impressive work, the authors investigated ultrasound-detectable CRTs daily in 1262 critically ill patients and demonstrated an incidence of 17% subclinical CRTs, 82% of which developed within 7 days after insertion.

Post-mortem studies on CICC-related vessel changes are scarce. In a case series from 1994 conducted by Raad et al., in which vessel wall changes were examined macroscopically and CICC biofilms were assessed by electron microscopy, a fibrin layer was noted on the external surface of all CICCs (*n* = 72), and mural thrombosis was detected in 38% of cases [10]. In 2003, Forauer et al. histologically examined six vessels with indwelling CICCs at autopsy and demonstrated severe pathology, including intimal injury, infiltration of media with acute inflammatory cells, and varying degrees of adherent thrombus [18]. In 2018, Wichmann et al. published data from 2012 and demonstrated a 38% prevalence of macroscopic catheter-related thrombosis in 61 human autopsies [19] Hence, the frequency of thrombus formation varies.

Since the data collection of the abovementioned studies, CICC materials have changed, and insertion techniques have improved, as real-time ultrasound guidance is now the only approved technique. Thus, the current prospective autopsy study was designed to further investigate any pathological vein wall changes associated with CICCs in modern healthcare.

## Methods

This prospective single-center observational study was approved by the Swedish Ethical Review Authority *(dnr 2018/866* and *06582–2019)*; the requirement for informed consent was waived by the Authority. The study was conducted at the Department of Genetics, Pathology and Molecular Diagnostics at Skåne University Hospital, Lund, Sweden. All deceased patients with a remaining CICC who were subject to autopsy at the department from December 2021 to October 2022 were included. The manuscript was written in accordance with the STROBE guidelines [20].

### Specimen preparation and microscopic examination

The autopsies and specimen collection were performed by one of the authors, assisted by an autopsy technician. The vein and the inserted CICC were carefully dissected from the proximal point of catheter insertion to the right atrium. A longitudinal midline incision was performed, exposing the internal part of the vessel. All pathological or suspicious macroscopic findings, such as thrombi, were documented. The specimens, including loose pathological findings, were pinned to a Styrofoam plate and fixed in a 4% formaldehyde solution. The specimens were fixed for a minimum of 24 h.

Transverse sections of 3–4 mm were subsequently cut at three predetermined vessel locations: the proximal portion (site of catheter insertion), the middle portion, and the distal portion (levelled at the tip of the CICC). All sections were dehydrated and embedded in paraffin blocks, followed by sectioning at 3 µm and staining with haematoxylin and eosin staining for all cellular structures, including thrombotic material and eosin-van Gieson staining for visualizing collagen, elastin, and smooth muscle. The slides were examined microscopically for histopathological changes.

### Outcomes

The main outcomes were macro- and microscopic changes observed at autopsy. The macroscopic outcome was thrombus (yes/no). The microscopic outcomes were: A) Thrombus adherent to the vessel wall (yes/no); B) Inflammation in the vessel wall (0: nothing visible, 1: minimal or a few discernible inflammatory cells subjacent to thrombus, 2: a modest number of inflammatory cells reaching deeper into the vessel wall, 3: marked cellular infiltration, with or without oedema, reaching throughout the vessel wall); and C) Fibrosis in the vessel wall (0: Nothing visible, 1: minimal, hardly discernible presence of fibroblasts or early collagen formation, 2: a mild collagenous thickening of the intima, 3: an obvious increase in collagen and intimal thickening).

### Data collection and statistical methods

Autopsy results and CICC- and patient-related data were extracted from the electronic health record. All data were inserted into a compiled, encrypted database (Excel, version 10, Microsoft, Santa Rosa, USA). The sample size was based on the number of available patients during the study period. The results are expressed as a number (%) for categorical variables and median (interquartile range) for continuous variables.

## Results

A summary of patient and catheter baseline data is shown in Table 1. In total, 12 autopsies on seven female (58%) and five male (42%) patients with CICCs were performed. The median age was 70 (63–76). The most common comorbidity was cardiovascular disease (75%), followed by suspected or diagnosed malignancy (58%), hypertension (42%), and hyperlipidaemia (42%). Eleven patients (92%) received routine thromboprophylactic enoxaparin 40 mg daily. One patient had severe coagulopathy and was without thromboprophylaxis. Two patients were on low-dose acetylsalicylic acid and none of the patients received other platelet inhibitors. No patient presented with a hypercoagulable profile based on routine coagulation tests during the length of stay. In total, 10 patients (83%) were treated with norepinephrine at some point in the catheter period, one with a low dose (≤ 0.1 μg.kg-1.min-1) and nine with high doses (> 0.1 μg.kg-1.min-1). The main causes of death were cardiac arrest secondary to acute myocardial infarction (25%), pneumonia and/or acute respiratory distress syndrome (25%), or hypovolaemic shock (17%).

All inserted catheters in the study were non-coated. All patients had at least one polyurethane catheter with different number of lumens, inserted. Two patients also had other CICCs in the same vessel; one patient with a silicone haemodialysis catheter and another patient with a PA-introducer & VA-ECMO cannula (Table 1). Most CICCs were 9.5 French (54%) and were inserted in the internal jugular vein (92%), with the majority on the right side (77%). The median number of days with a catheter was 7 (1.8–20). None of the patients had a CICC within one year prior to death, there were no immediate complications at insertion, and all insertions were performed with only 1 needle pass. Beyond CRT, patient 6 also had a thrombus in the right atrium around an ICD cable.

**Table 1:** Patient and catheter characteristics

**Patient characteristics**	**Patients, *n* = 12 (100%)**
**Gender; female**, number	7 (58%)
**Age** (years), median (interquartile range)	70 (63–76)
**BMI**, median (interquartile range)	26 (22–28)
**Length of hospital stay** (days), median (interquartile range)	8.5 (4.5–24)
**Comorbidities**, number	
*Cardiovascular disease*	9 (75%)
*Diagnosed or suspected malignancy*	7 (58%)
*Hypertension*	5 (42%)
*Hyperlipidaemia*	5 (42%)
*Lung disease (COPD, asbestosis)*	4 (33%)
*Smoking*	4 (33%)
*Diabetes*	3 (25%)
*Cerebrovascular disease*	3 (25%)
*Kidney failure (with or without dialysis)*	2 (17%)
*Alcoholism*	2 (17%)
*Psychiatric disorders (depression, anxiety)*	2 (17%)
*Heart failure*	1 (8.0%)
*Organ transplantation (kidney and pancreas)*	1 (8.0%)
*Obesity*	1 (8.0%)
*Previous deep venous thrombosis or pulmonary embolism*	0 (0.0%)
**Cause of death**, number	
*Acute myocardial infarction*	3 (25%)
*Pneumonia and/or acute respiratory distress syndrome*	3 (25%)
*Hypovolaemic shock*	2 (17%)
*Multiorgan failure*	1 (8.0%)
*Heart failure*	1 (8.0%)
*Septic shock*	1 (8.0%)
*Intracranial haemorrhage*	1 (8.0%)
**Patients with another CICC within one year before death**, number	0 (0.0%)
**Patients with prophylactic enoxaparin**, number	11 (92%)
**Patients with 75 mg acetylsalicylic acid daily**	2 (17%)
**Routine coagulation during length of stay**, median (interquartile range)	
*Platelet count (*× *10*^*9*^*/L)*	269 (146–301)
*Prothrombine time, INR*	1.2 (1.0–1.4)
*Acute partial thromboplastin time (s) (normal range 26–33 s)*	29 (27–34)
*Fibrinogen (g/L), n* = *10*	2.8 (1.8–5.4)
**Catheter characteristics**	**Catheters, *n* = 13 (100%)**^**a**^
**Type of catheter**, number	
*Non-coated polyurethane central venous catheter*^*b*^	12 (92%)
*Silicone central haemodialysis catheter*^*c*^	1 (8.0%)
**Catheter length**, number	
*15 cm*	8 (62%)
*20 cm*	5 (38%)
**Number of catheter lumen**, number	
*1 (5 French)*	1 (8.0%)
*2 (7 and 13.5*^*d*^* French)*	3 (23%)
*3 (7 French)*	2 (15%)
*5 (9.5 French)*	7 (54%)
**Insertion site**, number	
*Internal jugular vein*	12 (92%)
*Subclavian vein*	1 (8.0%)
**Insertion side; right**, number	10 (77%)
**Number of skin punctures**, number	1 [1]
**Real time ultrasound guided insertion**, number	12 (92%)
**Insertion complications**, number	0 (0.0%)
**Days with catheter**, median (interquartile range)	7 (1.8–20)

^a^ Two subjects had multiple centrally inserted central catheters (CICCs) inserted in the same vessel on the same day: one patient with polyurethane CICC and one silicone CICC and one patient with CICC, PA-introducer & VA-ECMO cannula. The latter is not reported in this table as they were inserted at the same day as death

^b^ Nine MeritMedical®, Careflow® and three Arrow® Blue FlexTip® CICCs

^c^ MedComp® Hemo-Cath®

^d^ Haemodialysis catheter

**Fig. 1:**
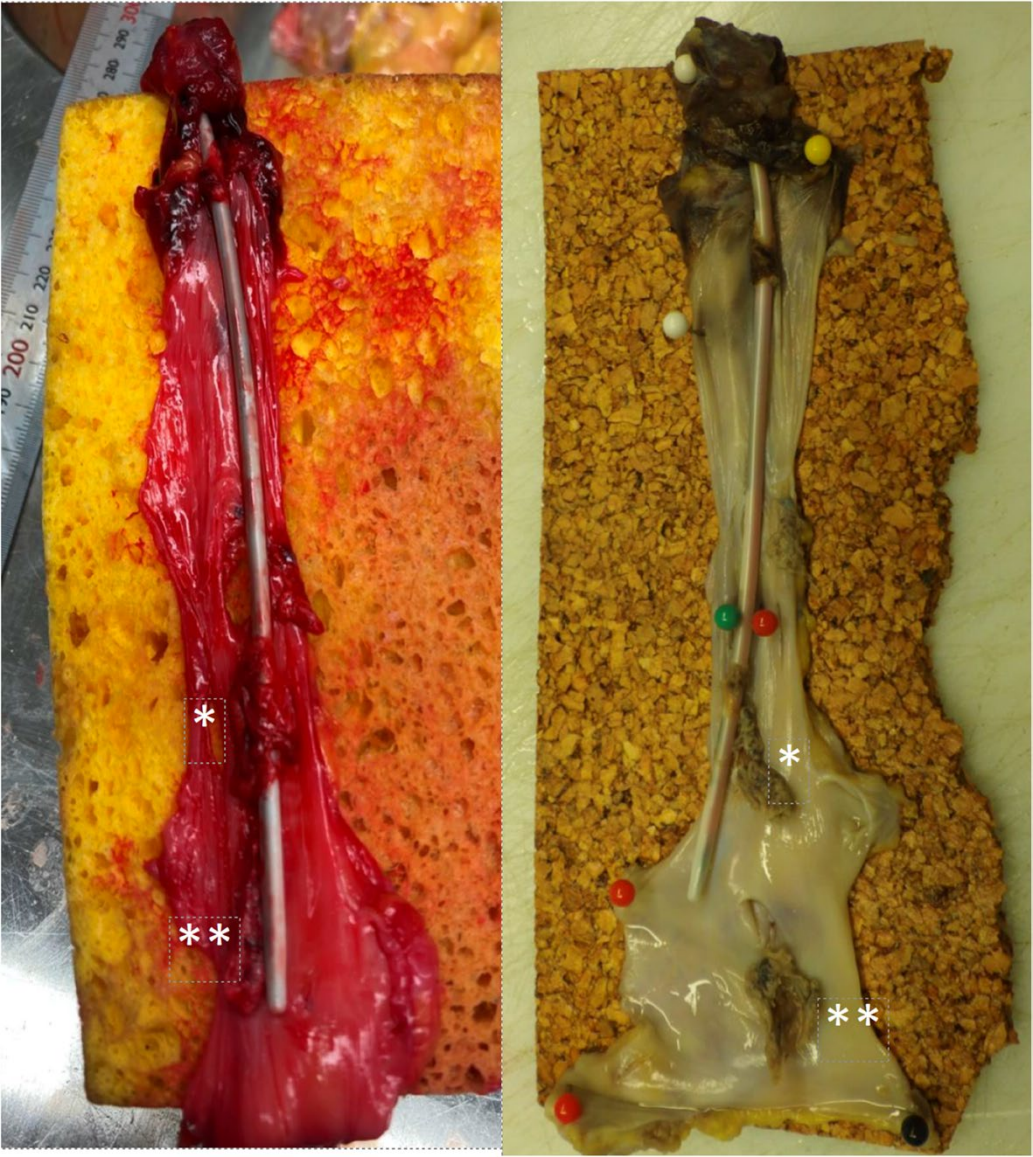
Macroscopically, thrombi attached to the centrally inserted central catheter (*) and to the adjacent vessel wall (**) were observed in all cases (100%). Photographs were taken before and after formalin fixation

**Fig. 2:**
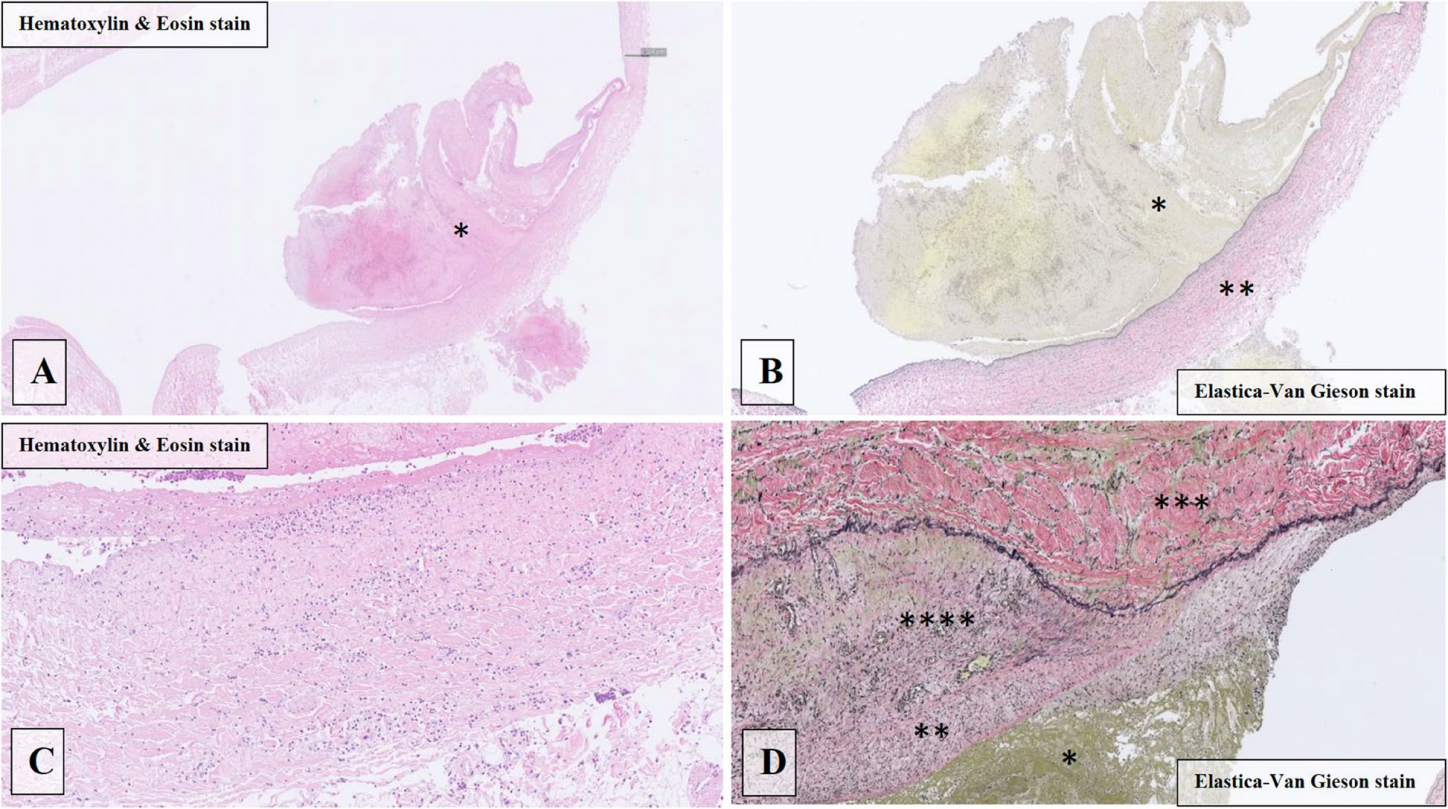
Microscopical changes observed at autopsy in the vein wall after central venous catheterization. Panels **A** and **B** show a large microscopic thrombus (*) adherent to the vessel wall. Marked inflammation (grade 3) with cellular infiltration and oedema reaching throughout the vessel wall can be observed (**). Panel **C** represents a normal vessel wall, which can be compared with Panel **D**, demonstrating a severe degree of fibrosis with extreme thickening of the intima (***) and a marked increase in mucosal collagen (**) subjacent to the adherent thrombus (*) and a severe degree of inflammation with transmural infiltration of inflammatory cells (****)

The macro- and microscopic changes observed are described in detail in Table 2. Thrombi were observed macroscopically and microscopically attached to the CICC, as well as to the adjacent vessel wall (mural thrombus), in all cases (100%); see examples in Figs. 1 and 2. Microscopically, varying degrees of inflammatory changes in vessel walls were observed in all cases, with varying degrees of fibrosis observed in eight cases (67%). As some thrombus material came loose in the dissection procedure, complete data on the exact location of all CRTs is not available. However, all cases had thrombi located in the stained section from the distal portion, i.e., close to or at the CICC tip.

**Table 2:** Macro- and microscopic changes observed in the vessel wall on autopsy

			**Thrombus**		**Vessel Wall Changes**	
**Days with central venous catheter**	**Multiple catheters, same vessel**	**Patient number**	**Macroscopic (Y/N)**	**Microscopic adherent to the vessel wall (Y/N)**	**Inflammation (0–3)**^**a**^	**Fibrosis (0–3)**^**b**^
1	No	1	Y	Y	3	0
1	CICC^c^, PA^d^-introducer & VA-ECMO^e^ cannula	6	Y	Y	2	3
1	No	7	Y	Y	1	1
2	No	12	Y	Y	1	0
3	No	2	Y	Y	2	2
7	No	8	Y	Y	1	0
7	No	9	Y	Y	1	0
12	No	3	Y	Y	3	2
19	No	5	Y	Y	3	2
22	CICC & CDC^f^	4	Y	Y	3	3
22	No	10	Y	Y	1	0
25	No	11	Y	Y	3	3

^a^ Degree of inflammation: 0 – nothing visible; 1 – minimal or a few discernible inflammatory cells subjacent to thrombus; 2 – modest number of inflammatory cells reaching deeper into vessel wall; 3 – marked cellular infiltration, with or without oedema, reaching throughout the vessel wall

^b^ Degree of fibrosis: 0 – nothing visible; 1 – minimal, hardly discernible presence of fibroblasts or early collagen formation; 2 – a mild collagenous thickening of the intima; 3 – an obvious collagen increase and intimal thickening

^c^ Centrally inserted central catheter

^d^ Pulmonary artery

^e^ Venoarterial extracorporeal membrane oxygenation

^f^ Silicone central dialysis catheter

## Discussion

This prospective observational autopsy study examined macro- and microscopic changes in vein walls with indwelling short-term CICCs. The results demonstrate thrombus formation and varying degrees of inflammatory changes in all cases, along with fibrosis in 67% of all investigated cases. Even if these results are not always clinically apparent, they are a cause of concern, considering their potential consequences, such as pulmonary embolism, catheter-related infections, and chronic venous stenosis. It should also be observed that all CICC-related thrombosis in the present study developed in patients without a pro-coagulable profile and that all patients were either given routine thromboprophylaxis or exhibited significant coagulopathy throughout the CICC period.

To the best of our knowledge, the only previous study that has evaluated macro- and microscopic venous changes of indwelling CICCs in human patients was published by Forauer et al. in 2003 [18]. {Forauer, 2003 #31} In a total of six specimens, thrombi with catheter-to-vein wall bridges were observed, with half of all patients presenting histological vessel wall changes equivalent to what we describe as grade 1–2 inflammation, whereas the other half presented changes corresponding to grade 3 inflammation (with grade 0–3 fibrosis) in the present study. Only minor inflammatory changes were seen in short-term catheters in the study by Forauer et al. (inserted for fewer than 14 days), indicating that severe vessel wall inflammation as well as fibrosis may be a direct consequence of long-term inflammatory changes [6]. In contrast, marked inflammatory and fibrotic changes in individuals with catheters in place for fewer than 14 days were observed in the study by Forauer et al., as also described in our material (Table 2), indicating that severe vessel wall changes can occur independently of the length of catheterization. Patient 6, with only one day with CICCs, presented with unexpected grade 3 fibrosis. It should be observed that this patient, beyond several large-bore catheters in the internal jugular vein also had thrombus in the right atrium along an ICD cable, which probably explains the histopathological findings (Table 2). This is an important observation however, and should raise concerns about whether multiple catheters should be inserted into the same vessel, as both the catheter-to-vessel ratio as well as multiple catheters inserted in a single vessel are known concerns regarding CRT, particularly when vessel assessment with ultrasound is not performed pre-procedurally or during the catheterization period [1, 21].

As described in the study by Wu et al., in which 1 262 critically ill patients were controlled for CRT daily using ultrasound and Doppler, the CRT incidence was 17% compared to 100% in the present study [5] – strengthening the argument of seeking for CRT in critically ill patients. It should be noted that although ultrasound/Doppler investigation is very good, the sensitivity/specificity for CRT is not 100%. Moreover, only the proximal part of the CICC vessel was investigated in the study by Wu et al. and given that most of the thrombi detected in the present study were located close to the CICC tip, ultrasound investigation of the proximal part of the vessel will not detect all CRTs. However, other studies have shown ultrasound to be helpful not only for CRT screening but also when assessing factors associated with the increased risk of CRT, such as catheter-tip positioning, which could help guide CICC-related decision-making [13, 21, 22].

In line with the present results, previous histological studies on animal models evaluating macro- and microscopic changes occurring with CICCs have demonstrated the formation of fibroblastic sleeves and thrombosis [23–25]. Further examination of the fibroblastic sleeve demonstrated a composition of smooth muscle cells and collagen covering a layer of endothelial cells, with presumed endothelial dysfunction and abnormal anticoagulatory function [24]. In the adjacent venous wall, intimal hyperplasia and inflammatory cell infiltration was observed with subsequent venous wall thickening [24, 26–28]. Moreover, the authors not only hypothesize that a catheter-related thrombosis can spread to form mural thrombi through catheter-to-vein wall bridges but also that the thrombi might lose support and detach from the catheter, causing embolism [8, 24]. These findings highlight the potential harm of CICC-related thrombi demonstrated in the present study.

Additionally, the inflammatory reaction caused by the CICC insertion – and hypothetically, the catheter type and material itself [29]—can lead to an inflammatory response with inflammatory vessel wall changes, similar to the ones demonstrated in 75% of the patients in this study. These changes include inflammatory cell infiltration with increased oxidative stress, which may activate leukocytes, releasing myeloperoxidase and activating the coagulation cascade [30]. This not only facilitates intramural thrombus formation but also induces smooth muscle cell hypertrophy, which may evolve into central venous stenosis, a finding most commonly reported in patients with large-bore dialysis catheters [6, 12]. Last, CICC-related thromboses are also associated with catheter-related infections and are known to cause CICC occlusion, leading to delays in treatment [1, 4, 31].

An important aspect of the current study is the recognition of study limitations. The sample size was based on the number of autopsies during the study period, meaning that further studies are needed to confirm the validity, reproducibility, and applicability of the results. Moreover, there is a risk of underestimating the worst pathological findings, as only a limited number of vessel wall sections were investigated and the contralateral side, serving as a control vessel, was not examined. This decision was based on previous results from autopsies of patients diagnosed with severe systemic COVID-19 disease [32], some of which with CICCs, where the venous system was specifically studied. No thrombus formation was found on the contralateral side in any case. It should also be noted that peripherally inserted central catheters were not included in the present study. Finally, there is a risk of selection bias, as it tends to be the sickest patients who are subject to autopsy, which means that the included patients may not be representative of a general patient cohort with CICCs. This is also exemplified by the fact that 54% of the patients had a 5-lm, 9.5 French CICC, which will create larger blood congestion than a thinner catheter and thus increase the risk of thrombus formation.

## Conclusions

In summary, this prospective observational study demonstrated that CRT is highly prevalent and that adjacent inflammatory vessel wall thickening also occurs frequently, despite a limited duration of CICC use. The consequences of these findings are important, as CRT may cause pulmonary embolisms, catheter-related infections, and chronic venous stenosis. Further research on these findings is crucial, as CICCs are indispensable in modern healthcare, and complications may be masked by the general disease that was the indication for CICC insertion.

## Data Availability

The datasets used and/or analysed during the current study are available from the corresponding author upon reasonable request.

## Abbreviations

CICC: Centrally inserted central catheters
CRT: Catheter-related thrombosis
